# Efficacy and safety of anlotinib with and without EGFR-TKIs or immunotherapy in the treatment of elder patients with non-small-cell lung cancer: a retrospective study

**DOI:** 10.1186/s12890-022-01981-5

**Published:** 2022-05-06

**Authors:** Wenxian Wang, Lan Shao, Yibing Xu, Zhengbo Song, Guangyuan Lou, Yiping Zhang, Ming Chen

**Affiliations:** 1 The Second Affiliated Hospital of Soochow University, Suzhou, China; 2 Department of Medical Oncology, The Cancer Hospital of the University of Chinese Academy of Sciences (Zhejiang Cancer Hospital), Hangzhou, China; 3 Institute of Cancer and Basic Medicine(IBMC), Chinese Academy of Sciences, Hangzhou, China; 4 Department of Radiotherapy Oncology, The Second Affiliated Hospital of Soochow University, 1055 Sanxiang Road, Suzhou, 215004 Jiangsu Province China

**Keywords:** Non-small cell lung cancer, Prognosis, Angiogenesis, Tyrosine kinase inhibitors

## Abstract

**Background:**

Anlotinib is a multitarget tyrosine kinase inhibitor for treating patients with advanced non-small cell lung cancer (NSCLC). We aimed to assess the efficacy and safety of anlotinib in elder patients with advanced NSCLC.

**Methods:**

Elder patients with advanced NSCLC who received anlotinib were enrolled. They were all age ≥ 65 years and with demonstrated records of EGFR gene status. All patients had received treatment with anlotinib or immune checkpoint inhibitors (ICIs)/EGFR-TKIs. The efficacy was evaluated according to the efficacy evaluation criteria for solid tumors (RECIST 1.1). Common Adverse Events Evaluation Criteria (CTCAE 4.03) were used to evaluate adverse drug reactions.

**Results:**

A total of 91 patients were included in this study. We divided the patients into two groups (EGFR wild type: 60 patients; EGFR mutation: 31 patients). Among EGFR negative patients, the progression-free survival (PFS) for anlotinib monotherapy and anlotinib combination ICI therapy was 3.2 months and 5.0 months, respectively (*P* = 0.012). The difference in overall survival (OS) between monotherapy and combination therapy was also significant (9.5 vs. 18.4 months, respectively *P* = 0.010). Interestingly, we further analyzed differences between patients with hypertension and without hypertension, and found that hypertension was associated with better prognosis (5.7 vs. 1.4 months, *P* < 0.0001). In the EGFR mutation group, the PFS for anlotinib and EGFR-TKI combination treatment indicated better efficacy than that of anlotinib monotherapy (1.83 months vs. 7.03 months, respectively, *P* = 0.001). The median OS for monotherapy and combination therapy in the EGFR mutation group showed no statistical difference (28.34 months vs. 31.37 months, *P* = 0.223). The most common adverse reactions were hypertension, fatigue, and hand-foot syndrome, mainly of grade 1 or 2. No significant increase in adverse reactions was observed in patients  ≥ 70 years of age.

**Conclusions:**

Anlotinib treatment and combination regimens resulted in good efficacy and controllable adverse reactions in elder patients with advanced NSCLC.

## Background

Lung cancer is the leading cause of cancer-related deaths worldwide [1]. Currently, targeted therapy and immunotherapy can prolong survival in patients with advanced non-small cell lung cancer (NSCLC) [2–4]. However, choosing a treatment plan is difficult for patients who have failed more than second-line treatment. Most patients with advanced NSCLC can receive only single chemotherapy in clinical practice. Some patients cannot tolerate chemotherapy and receive only single-agent immunotherapy, with limited effects. More effective treatment regimens in clinical practice remain lacking.

Anti-angiogenic drugs have effects in advanced NSCLC [5]. The anti-VEGF monoclonal antibody bevacizumab prevents the binding of the VEGF ligand to its receptor and has been demonstrated to increase efficacy and survival in patients with NSCLC [6]. In addition, apatinib, another anti-angiogenic drug, has the potential to improve efficacy in patients with advanced NSCLC [7, 8]. The new oral tyrosine kinase inhibitor anlotinib inhibits the VEGF receptor, fibroblast growth factor receptor, platelet-derived growth factor receptors and stem cell factor receptor [9]. A phase I study has reported that anlotinib is generally well tolerated in patients with advanced drug-resistant solid tumors, with a daily oral dose of 12 mg or lower (10 mg and 8 mg) [9]. In a phase III trial, anlotinib, compared with placebo, improved the overall survival (OS) for refractory advanced NSCLC, and its toxicity was controllable [10]. Therefore, anlotinib was approved by the China Food and Drug Administration in 2018 as a third-line treatment for refractory advanced NSCLC.

Owing to low immunity and the presence of many heart, lung, and kidney diseases, the pharmacokinetic behavior of drugs in elder patients with advanced NSCLC differ from those in younger patients. In addition, elder patients need more safe drugs as first choice treatments. Therefore, the efficacy and safety of anlotinib in the treatment of advanced NSCLC in elder patients is a clinical issue worthy of discussion. In the ALTER0303 trial, a subgroup analysis of 28 patients elder than 70 years, 16 of whom were treated with anlotinib, has also confirmed better progression-free survival (PFS) for anlotinib than placebo in elder patients (*P* < 0.0028) [11]. However, because of the many limiting factors in these studies, such as the clinical trial samples used, further exploration of the efficacy and safety of anlotinib is necessary, particularly for anlotinib in combination with other treatments such as immunotherapy, in elder patients in the real world.

No independent study has been conducted on the use of anlotinib in the treatment of advanced NSCLC in elder patients. Therefore, this study intended to explore the efficacy and safety of anlotinib alone or together with other drugs in elder patients with advanced NSCLC, to provide new evidence supporting the use of anlotinib in the treatment of these patients.

## Materials and methods

### Patient characteristics

We conducted a retrospective study of patients with stage IIIB/IV advanced NSCLC. The histologic classification of NSCLC was based on the World Health Organization criteria (2015 version). Patients who met the following inclusion criteria were included in the study: clinicopathological information recorded, including smoking history, age, sex, stage, and histological type of NSCLC; age ≥ 65 years; pathologic examination of tumor specimens performed with demonstrated records of EGFR/ALK gene status; and treatment regimens including anlotinib. The exclusion criteria included: missing clinical data, including age, sex, and stage; pathologic examination indicating small cell lung cancer; and treatment regimens of anlotinib combined with chemotherapy. We compared the general characteristics of the two groups of patients with or without EGFR mutation. In addition, we explored the effects of Kirsten rat sarcoma viral oncogene homolog (KRAS) gene mutation on anlotinib treatment, particularly anlotinib in combination with immunotherapy.

### Treatment and response assessments

We collected data on patients with NSCLC during anlotinib treatment. Anlotinib was taken orally on days 1–14 (once per day) in a 21-day cycle, and the specific doses (12, 10, and 8 mg) were accurately recorded according to clinician selection after patient evaluation. Clinicians determined the initial dosage of anlotinib according to patient condition. Some patients who participated in the clinical trials received anlotinib combination as a second-line treatment, and three patients participated in clinical trials. A dose reduction or withdrawal was allowed. Because this was a retrospective study, the treatment modes comprised not only anlotinib monotherapy but also combination treatments with immune checkpoint inhibitors (ICIs) or EGFR-TKIs. The EGFR-TKIs used in the initial treatment and those used in combination with anlotinib were the same. All ICIs and targeted regimens were administered in standard doses, on the basis of NCCN guidelines. Response was assessed according to the RECIST v1.1 criteria. Before analysis, efficacy was examined by two oncologists who evaluated the tumor response according to RECIST 1.1 criteria on the basis of chest CT and/or brain MRI every 4–8 weeks.

### Evaluation of efficacy and prognosis

Tumor response efficacy was determined as complete response (CR), partial response (PR), stable disease (SD), or progressive disease (PD). The overall response rate (ORR) indicated CR and PR. The disease control rate (DCR) indicated CR, PR, and SD. PFS was defined as the time from the first day of anlotinib treatment to disease progression. The OS was the time from the date on which advanced NSCLC was confirmed to the date of mortality or the last follow-up. Toxicity was recorded and assessed according to Common Terminology Criteria for Adverse Events (CTCAE) version 4.03.

### Statistical analysis

χ^2^ tests were used for the comparison of the distributions of cohort characteristics and treatment types between the EGFR mutation and EGFR wild type group. Kaplan–Meier estimates and log-rank tests were used to evaluate PFS and OS, respectively. In addition, we performed a series of Cox proportional hazard regression analyses to determine the factors independently associated with PFS and OS. All statistical analyses were performed in SPSS (version 25.0; SPSS, Inc., Chicago, IL, USA). Two-sided *P* values < 0.05 were considered statistically significant. The last follow-up date was May 10, 2021.

## Results

### Patient characteristics

In this study, we enrolled 91 elder patients with advanced NSCLC. We considered that the treatment regimens and prognosis of patients with EGFR mutation differed from those of EGFR negative patients. Then, only one patient was with ALK positive. We divided the patients into two groups according to whether the EGFR gene was mutated. The baseline characteristics of the patients are summarized in Table 1. A total of 50 adenocarcinomas and 41 non-adenocarcinomas were found. Twenty-seven patients had a history of smoking, and 64 had never smoked. There were 68 men and 23 women, with an average age of 69 years (range 65–84 years). Among them, 31 had EGFR mutation, and 60 had wild type EGFR; 16 patients had exon 19 deletions, nine patients had exon 21 L858R, and the remaining six patients had rare EGFR mutations. In addition, five patients had KRAS mutations (8.3%) in the EGFR negative group. Nine patients were treated with anlotinib as the first-line treatment because they could not tolerate chemotherapy; 11 of 91 patients had liver metastases (12.1%), and eight patients had brain metastases (8.8%). A total of 84 patients (92.3%) had a PS score of 0–1, and seven patients (7.7%) had a PS score ≥ 2.

**Table 1:** Baseline characteristics stratified by EGFR gene status in advanced NSCLC

Characteristics	Total (n = 91)	EGFR negative (n = 60)	EGFR mutation (n = 31)
*Age*			
Median (range)	69 (65–84)	69 (65–84)	68 (65–77)
*Sex*			
Male	68 (74.7%)	53 (88.3%)	15 (48.4%)
Female	23 (25.3%)	7 (11.7%)	16 (51.6%)
*Smoking history*			
Never smoking	27 (29.7%)	9 (15.0%)	18 (58.1%)
Ever smoking	64 (70.3%)	57 (85.0%)	13 (41.9%)
*ECOG PS*			
0–1	84 (92.3%)	57 (95.0%)	27 (87.1%)
2	7 (7.7%)	3 (5.0%)	4 (12.9%)
*Histological*			
Adenocarcinoma	50 (54.9%)	21 (35.0%)	29 (93.5%)
Others	41 (45.1%)	39 (65.0%)	2 (6.5%)
*Liver metastases*			
No	80 (87.9%)	51 (85.0%)	29 (93.5%)
Yes	11 (12.1%)	9 (15.0%)	2 (6.5%)
*Brain metastases*			
No	83 (91.2%)	56 (93.3%)	27 (87.1%)
Yes	8 (8.8%)	4 (6.7%)	4 (12.9%)
*KRAS gene status*			
Mutation	5 (5.5%)	5 (8.3%)	0
Negative	24 (26.4)	14 (23.3%)	10 (32.3%)
Unknown	62 (68.1%)	41 (68.4%)	21 (67.7%)
*Anlotinib treatment modes*			
Monotherapy	56 (61.5%)	38 (63.3%)	18 (58.1%)
Combination	35 (38.5%)	22 (36.7%)	13 (41.9%)
*Lines of Anlotinib treatment*			
First-line	9 (9.9%)	8 (13.3%)	1 (3.2%)
≥ Second-line	82 (90.1%)	52 (86.7%)	30 (86.8%)

### Efficacy and treatment prognosis in the EGFR negative group

**Fig. 1:**
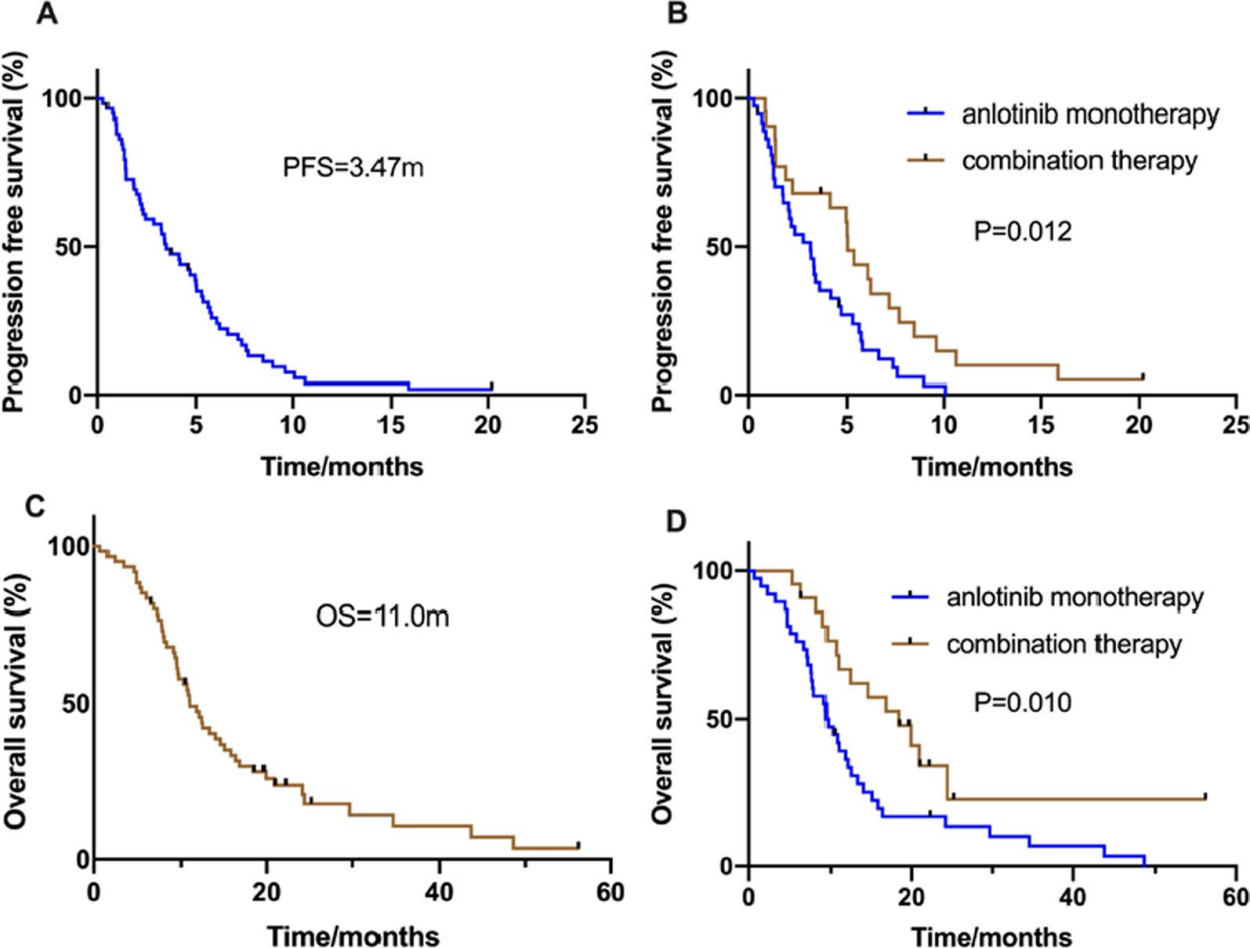
Kaplan Meier estimates of progression-free survival (PFS) and overall survival (OS) according to type of anlotinib treatment modes in patients with EGFR negative **A** PFS in all EGFR negative NSCLC patients; **B** PFS in the anlotinib monotherapy and combination therapy groups (*P* = 0.012); **C** OS in all EGFR negative NSCLC patients; **D** OS in the anlotinib monotherapy and combination therapy groups (*P* = 0.010)

In the EGFR negative group, the efficacy data for anlotinib were as follows: CR, n = 0; PR, n = 9; SD, n = 40; and PD, n = 11. The ORR was 15.0% and the DCR was 81.7%. The median PFS (mPFS) was 3.47 months (95% CI 2.439–4.495; Fig. 1A). A total of 38 cases were treated with single-agent anlotinib, and 22 were treated with ICIs combined with anlotinib. The ORR for monotherapy was 13.2% and the DCR was 76.3%. The ORR for combination therapy was 18.2% and the DCR was 90.9%. The mPFS was estimated to be 3.2 months for patients receiving anlotinib monotherapy and 5.0 months for patients receiving anlotinib combined with ICIs (*P* = 0.012, Fig. 1B). According to the KRAS gene status, we further analyzed the PFS for anlotinib treatment in patients with KRAS mutation. For the five patients with KRAS mutation, the PFS was 7.6 months with anlotinib monotherapy and 6.2 months with anlotinib combined with ICIs.

The results of univariate analysis of PFS indicated that the PFS was not associated with sex (*P* = 0.968), smoking history (*P* = 0.920), pathological subtype (*P* = 0.302), PS (*P* = 0.134), brain metastasis (*P* = 0.262), and the line use of anlotinib (*P* = 0.740). However, liver metastasis (*P* = 0.016) and the anlotinib treatment mode (*P* = 0.014) were independent factors that influenced PFS. Subsequent multivariate analysis showed that the treatment mode of anlotinib (*P* = 0.004) was an independent factor influencing PFS. The details of the single-factor and multi-factor analysis of PFS are shown in Table 2.

**Table 2:** Univariate and multivariate analysis of progression free survival (PFS) in EGFR gene negative NSCLC patients

	Univariate analysis		Multivariate analysis	
	*P* value	95% CI	*P* value	HR (95% CI)
Sex	0.968	0.456–2.269	0.384	0.086–2.574
Smoking	0.920	0.502–2.145	0.383	0.108–2.351
ECOG PS	0.134	0.757–8.083	0.124	0.748–11.014
Histological	0.302	0.436–1.294	0.080	0.299–1.072
Liver metastases	0.016	1.200–5.966	0.061	0.961–5.597
Brain metastases	0.262	0.640–5.156	0.835	0.239–3.181
Anlotinib treatment modes	0.014	0.270–0.862	0.004	0.182–0.731
Lines of Anlotinib treatment	0.740	0.873–1.944	0.901	0.427–2.628

The median OS (mOS) for all 60 patients was 11.0 months (95% CI 8.776–13.224; Fig. 1C). The mOS was 9.5 months for patients receiving anlotinib monotherapy and 18.4 months for those receiving anlotinib combined with ICIs (*P* = 0.010, Fig. 1D). In addition, KRAS mutated patients had an OS of 9.5 months for anlotinib, and 21.0 months for anlotinib combined with ICIs (*P* = 0.364). According to the multivariate analysis of OS, PS (*P* = 0.005) and the lines of anlotinib treatment (*P* = 0.001) were independent factors affecting the prognosis.

### Efficacy and prognosis of treatments in the EGFR mutation group

**Fig. 2:**
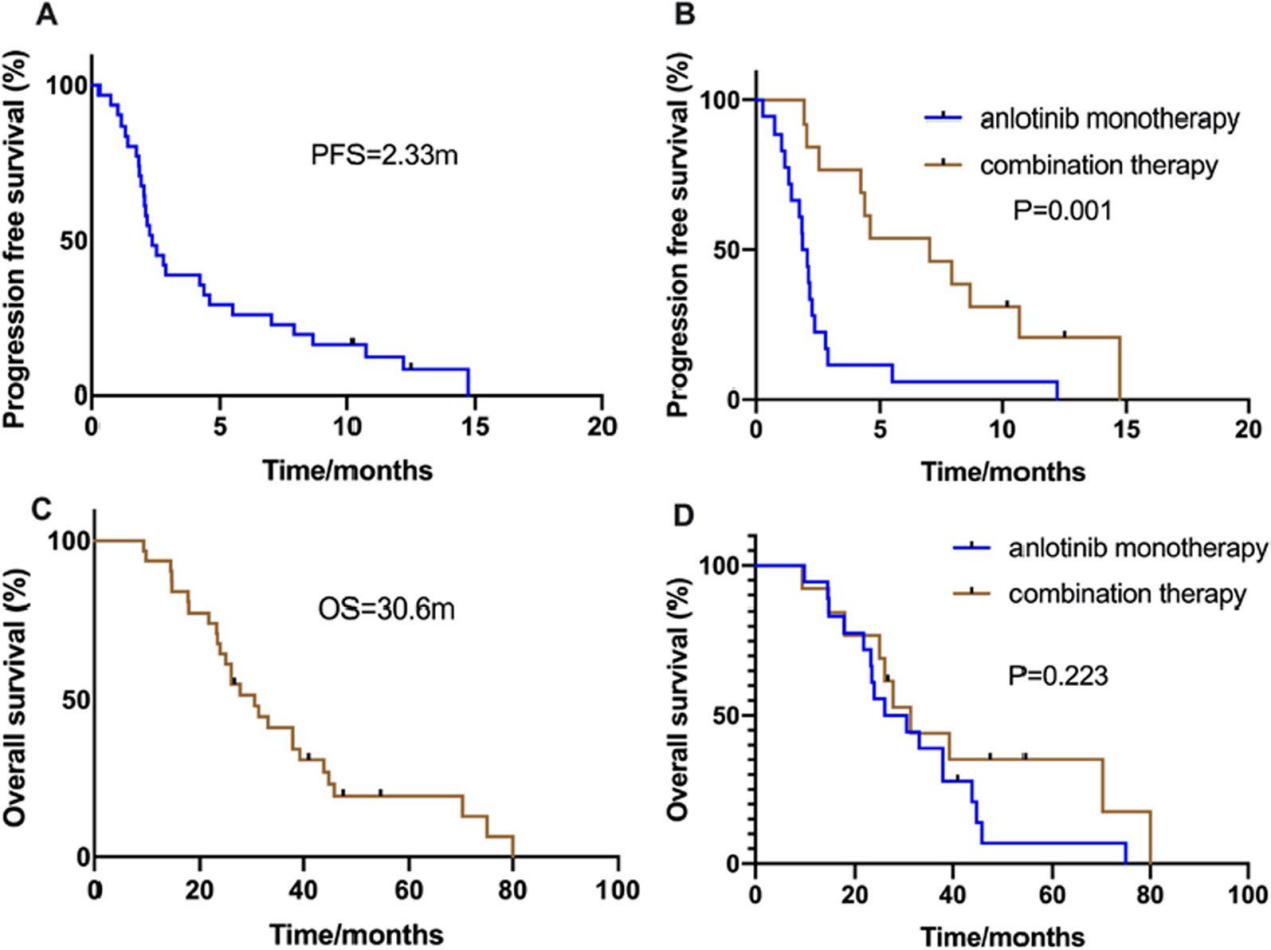
Kaplan Meier estimates of progression-free survival (PFS) and overall survival (OS) according to type of anlotinib treatment modes in patients with EGFR mutations **A** PFS in all EGFR mutated NSCLC patients; **B** PFS in the anlotinib monotherapy and combination therapy groups (*P* = 0.001); **C** OS in all EGFR mutated NSCLC patients; **D** OS in the anlotinib monotherapy and combination therapy groups (*P* = 0.223)

In patients with EGFR mutations, the efficacy of anlotinib was as follows: CR, n = 0; PR, n = 4; SD, n = 23; and PD, n = 4. The ORR was 12.9% and the DCR was 87.1%. The mPFS was 2.33 months (95% CI 1.570–3.097; Fig. 2A). A total of 18 patients were treated with single-agent anlotinib, 13 received combined treatment (nine receiving combined treatment with anlotinib and the original EGFR-TKI drugs, and four receiving immune checkpoint inhibitors combined with anlotinib). In monotherapy, the ORR was 0%, and the DCR was 77.8%. The ORR for combination therapy was 30.8% and the DCR was 100%. The PFS for monotherapy and combination therapy was 1.83 months and 7.03 months, respectively (*P* = 0.001, Fig. 2B). Multivariate analysis of PFS indicated that liver metastasis (*P* = 0.006), brain metastasis (*P* = 0.004), the PS score (*P* = 0.011), and anlotinib treatment mode (*P* = 0.002) were independent influencing factors.

The mOS for all 31 patients was 30.6 months (95% CI 23.894–37.306; Fig. 2C). The mOS for monotherapy and combination therapy was 28.34 months and. 31.37 months, respectively (*P* = 0.223, Fig. 2D). Multivariate analysis based on OS indicated that brain metastasis (*P* = 0.023) was an independent factor affecting prognosis.

### Safety and correlation with anlotinib efficacy

All patients were evaluated for toxicity, and five patients discontinued anlotinib treatment. Seven patients required decreased doses because of adverse reactions. Adverse reactions were recorded according to EGFR gene status and the mode of anlotinib treatment, and no significant differences were observed. The adverse effects are listed in Tables 3 and  4. The most common adverse reactions were hypertension, fatigue, loss of appetite, hand-foot syndrome, abnormal thyroid function, and diarrhea. Mainly grade 1 or 2 adverse reactions were observed, and fewer grade 3 or 4 adverse reactions were seen. No deaths were caused by anlotinib treatment, and the tolerability among elder patients was similar to that in younger patients, according to previous clinical data.

**Table 3:** Treatment-related adverse events with anlotinib treatment in EGFR negative NSCLC patients

Type	Anlotinib monotherapy (n = 38)		Anlotinib with ICIs (n = 22)	
	Grade 1–2	Grade 3–4	Grade 1–2	Grade 3–4
Hand-foot syndrome	16(42.1%)	2(5.3%)	13(29.0%)	2(9.0%)
Hypertension	17(44.7%)	2(5.3%)	12(54.5%)	1(4.5%)
Fatigue	11(28.9%)	1(2.6%)	8(36.4%)	1(4.5%)
Diarrhea	10(26.3%)	1(2.6%)	7(31.8%)	2(9.0%)
Anorexia	10(26.3%)	0	6(27.3%)	0
Abnormal liver function	9(23.7%)	0	6(27.3%)	0
Proteinuria	9(23.7%)	2(5.3%)	5(22.7%)	1(4.5%)
Mucositis oral	7(18.4%)	1(2.6%)	3(13.6%)	1(4.5%)
Thyroid dysfunction	6(15.8%)	0	3(13.6%)	0
Arthralgia	6(15.8%)	0	3(13.6%)	0
Headache	5(13.2%)	0	2(9.0%)	0

**Table 4:** Treatment-Related Adverse Events with anlotinib treatment in EGFR mutated NSCLC patients

Type	Anlotinib monotherapy (n = 18)		Anlotinib with ICIs (n = 13)	
	1–2级	3–4级	1–2级	3–4级
Hand-foot syndrome	7(38.9%)	1(5.6%)	4(30.8%)	1(7.7%)
Hypertension	7(38.9%)	1(5.6%)	5(38.5%)	1(7.7%)
Fatigue	6(33.3%)	1(5.6%)	4(30.8%)	2(15.4%)
Diarrhea	6(33.3%)	1(5.6%)	4(30.8%)	1(7.7%)
Anorexia	5(27.8%)	1(5.6%)	5(38.5%)	0
Abnormal liver function	4(22.2%)	0	4(30.8%)	0
Proteinuria	5(27.8%)	1(5.6%)	3(23.1%)	0
Mucositis oral	7(18.4%)	1(2.6%)	3(23.1%)	0
Thyroid dysfunction	3(16.7%)	0	1(7.7%)	0
Arthralgia	2(11.1%)	0	2(15.4%)	0
Headache	2(11.1%)	0	1(7.7%)	0

**Fig. 3:**
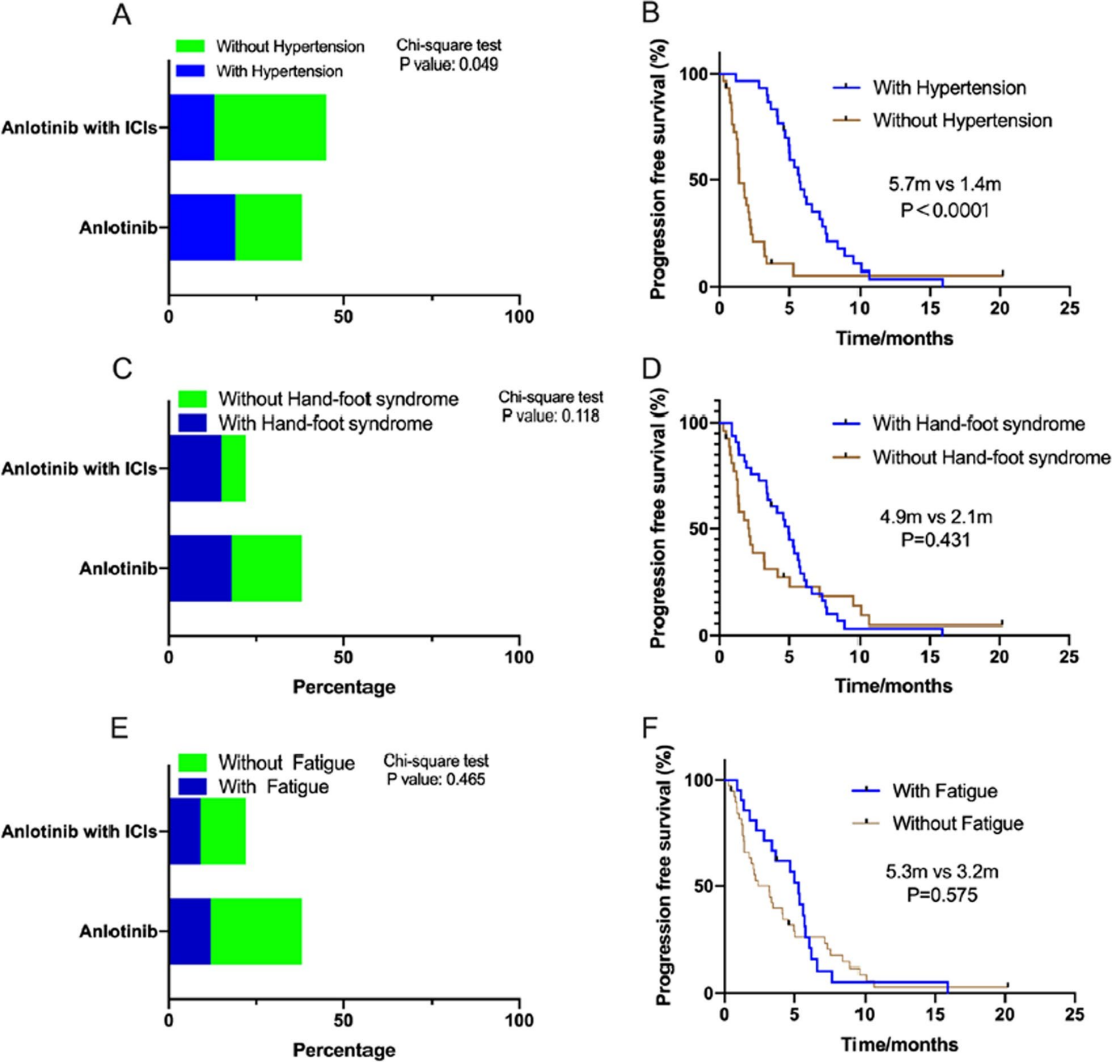
The comparison of the ration of the patients with the common adverse reactions (hypertension, hand-foot syndrome, and fatigue) and without them during anlotinib and combined with ICIs treatment in EGFR gene negative NSCLC. Then, stratification analysis of PFS of anlotinib between patients with and without the common adverse reactions. **A** The ratio of the patients with hypertension between anlotinib and combined with ICIs treatment was 50% and 58.9%, respectively (*P* = 0.049). **B** PFS of patients with and without hypertension in EGFR gene negative NSCLC was 5.7 months and 1.4 months (*P* < 0.0001). **C** The ratio of the patients with hand-foot syndrome between anlotinib and combined with ICIs treatment was 47.4% and 38%, respectively (*P* = 0.118). **D** PFS of patients with and without hand-foot syndrome was 4.9 months and 2.1 months (*P* = 0.431). **E** The ratio of the patients with fatigue between anlotinib and combined with ICIs treatment was 31.5% and 40.9% (*P* = 0.465). **F** PFS of patients with and without fatigue was 5.3 months and 3.2 months (*P* = 0.575)

**Fig. 4:**
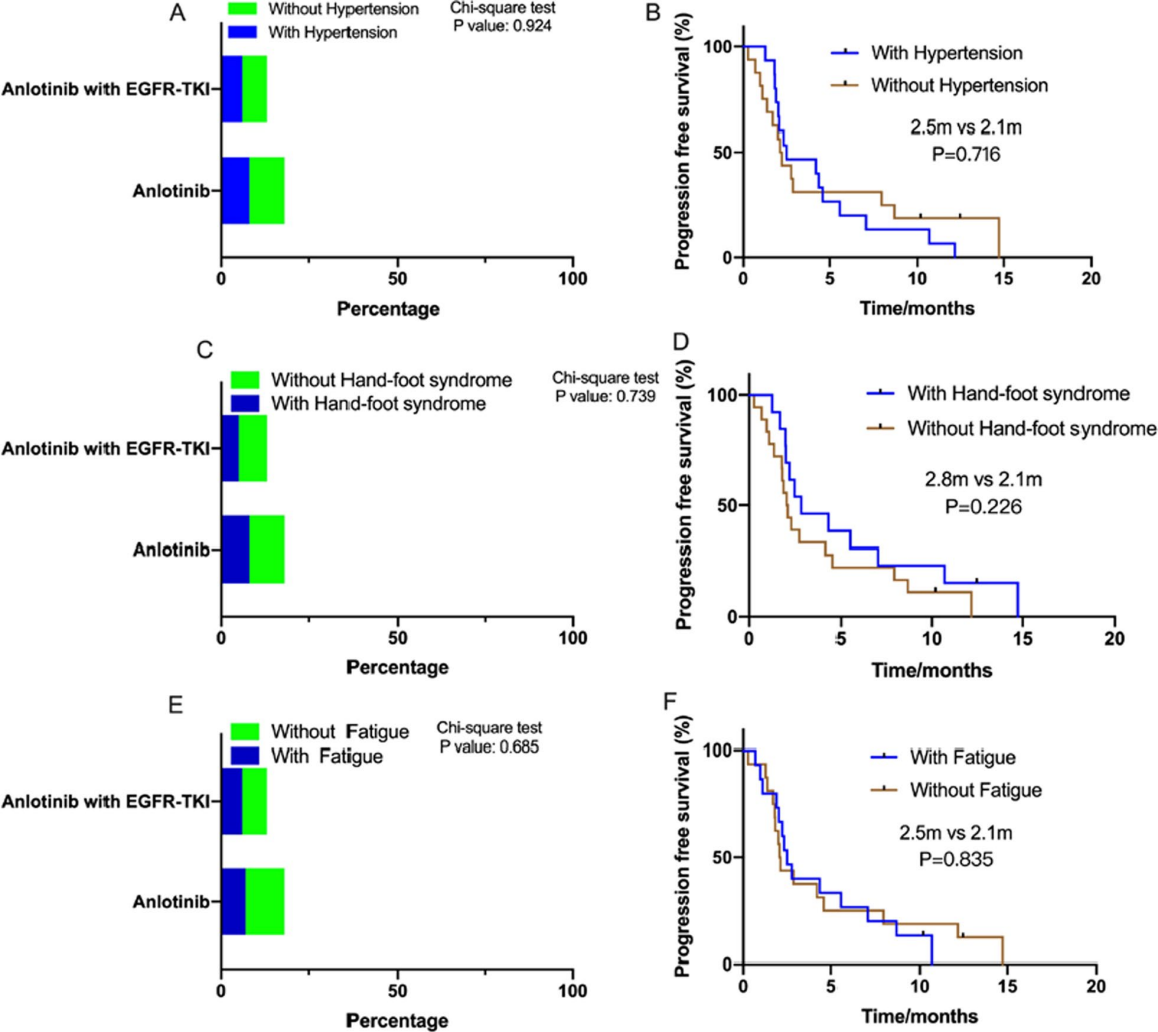
The comparison of the ration of the patients with the common adverse reactions (hypertension, hand-foot syndrome, and fatigue) and without them during anlotinib and combined with ICIs treatment in EGFR mutated NSCLC. Stratification analysis of PFS of anlotinib between patients with and without the common adverse reactions. **A** The ratio of the patients with hypertension between anlotinib and combined with ICIs treatment was 44.5% and 46.2%, respectively (*P* = 0.924). **B** PFS of patients with and without hypertension in EGFR mutated NSCLC was 2.5 months and 2.1 months (*P* = 0.716). **C** The ratio of the patients with hand-foot syndrome between anlotinib and combined with ICIs treatment was 44.5% and 38.5%, respectively (*P* = 0.739). **D** PFS of patients with and without hand-foot syndrome was 2.8 months and 2.1 months (*P* = 0.226). **E** The ratio of the patients with fatigue between anlotinib and combined with ICIs treatment was 38.9% and 46.2% (*P* = 0.685). **F** PFS of patients with and without fatigue was 2.5 months and 2.1 months (*P* = 0.835)

In addition, we analyzed the incidence of the most common adverse events (hypertension, hand-foot syndrome, and fatigue) and their relevance to anlotinib treatment, as shown in Figs. 3 and 4, according to EGFR gene status. The incidence of hypertension was 50% (19/38), and PFS significantly differed between patients with and without hypertension during anlotinib treatment in the EGFR gene negative group (Fig. 3B). However, we did not observe similar results in patients with EGFR mutation (Fig. 4B). The PFS of patients with hand-foot syndrome and fatigue was not longer than to that of patients without hand-foot syndrome and fatigue (Figs. 3D, E, 4D, E).

**Fig. 5:**
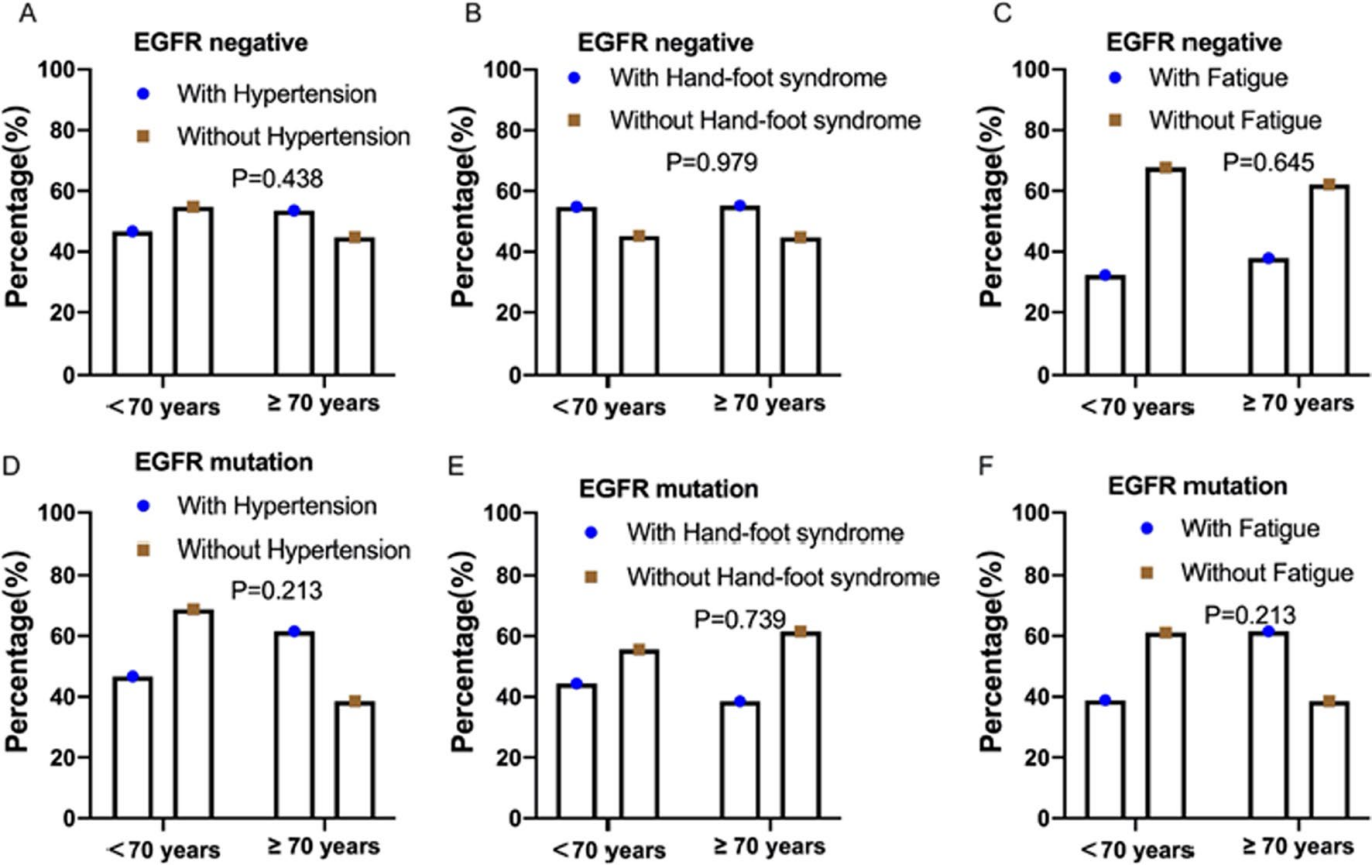
Compared the incidence of the common adverse events (hypertension, hand-foot syndrome, and fatigue) between the ages of ≥ 70 years and < 70 years according to EGFR gene negative and mutation groups. **A–C** There was no statistical difference in the proportion of EGFR-negative patients who experienced the common adverse events at age ≥ 70 years and < 70 years. **D–F** There was also no statistical difference in the proportion of EGFR-mutation patients

Furthermore, we compared the differences in adverse reactions in patients  ≥ 70 and < 70 years of age. The incidence of hypertension, hand-foot syndrome, and fatigue was compared in detail between the EGFR mutation and EGFR negative groups (Fig. 5). Patients ≥ 70 years of age did not show a greater incidence of major adverse reactions after anlotinib treatment than patients < 70 years of age, and treatments were well tolerated in elder patients with NSCLC.

## Discussion

This study evaluated the clinical benefit and safety of anlotinib as a treatment for elder patients with advanced NSCLC. Anlotinib had a good therapeutic effect in elder patients with advanced NSCLC, conferred advantages in combination therapy, and was well tolerated.

At present, the main treatments for advanced lung cancer include chemotherapy, targeted therapy, and immunotherapy. Combination therapy may be a future therapeutic direction. Therefore, choosing a treatment plan for these patients is important. Little evidence regarding the efficacy and safety of anlotinib in elder patients with NSCLC has been reported. ALTER 0303 subgroup analysis has shown that anlotinib is safe and tolerable among elder patients with lung cancer (≥ 70 years of age) [11]. Similarly, anlotinib has been found to be effective as a third-line treatment for elder patients with advanced lung cancer, with a disease control rate reaching 81.40%. Therefore, it is a good treatment choice for elder patients with advanced lung cancer after failure of multi-line therapy.

In this study, in patients with EGFR mutations, targeted therapies that can prolong survival and maintain quality of life were identified. However, almost all patients will develop resistance. Therefore, how to overcome or delay drug resistance is a major research topic. Elder patients require effective drugs with low toxicity. We found that the PFS for anlotinib monotherapy and anlotinib combination therapy was 1.83 months and 7.03 months, respectively (*P* = 0.001). Li et al. [12] have found that treatment with anlotinib combined with first-generation EGFR-TKIs can overcome resistance to EGFR-TKIs. In 20 patients with advanced NSCLC and in vitro experiments, the mPFS reached 15.7 months. Similarly, in vitro cell experiments indicated that anlotinib combined with gefitinib effectively inhibits the growth and cloning ability of lung cancer cells. Therefore, anlotinib combined with EGFR-TKIs may be able to reverse EGFR-TKI resistance to some extent, thus improving therapeutic effects in some patients. This treatment is also tolerated by elder patients. Therefore, different types of drug combinations may provide promising treatment options for patients with EGFR-TKI resistance.

For EGFR-negative patients with NSCLC, chemotherapy, ICIs, or a combination thereof comprise the first-line treatment regimens. However, some elder patients cannot tolerate chemotherapy, and may receive drug monotherapy or ICIs in combination with antiangiogenic drugs as the first-line treatment. Although anlotinib was approved as a third-line treatment for patients with NSCLC, with the development of research, the combination of immune and anti-vascular treatments is a major treatment focus. Chu et al., in a prospective study exploring the efficacy of sintilimab combined with anlotinib as a first-line therapy in 22 patients [13], have found an ORR of 72.7% and an mPFS of 15.0 months. Therefore, sintilimab plus anlotinib is a novel regimen for patients with advanced NSCLC. Moreover, Wang et al. have retrospectively enrolled 67 patients with advanced NSCLC receiving anti-PD-1 combined with anlotinib, and found an ORR of 28.4% and mPFS of 6.9 months [14]. The median age in this study was 60 years (range: 33–77 years). We further compared the difference between single-agent anlotinib therapy and combination therapy in elder patients with NSCLC and found an mPFS for anlotinib of 3.47 months. A total of 38 patients were treated with single-agent anlotinib, and 22 received combined therapy. The PFS for monotherapy and combination therapy was 3.2 months and 5.0 months, respectively (*P* = 0.012). The results of multivariate analysis also demonstrated that the anlotinib treatment mode (*P* = 0.004) was an independent influencing factor for PFS. Zhang et al. have conducted a retrospective analysis of 177 patients with non-small cell lung cancer [15]. In the first-line treatment and the second-line and later-line treatments, the PFS for anlotinib combined with immunotherapy was longer than that for anlotinib monotherapy, and the security of anlotinib was acceptable. Therefore, combined therapy can improve the treatment efficacy, including that in elder patients, and may become a new treatment model in the future.

KRAS is one of the most commonly mutated oncogenes in NSCLC. The incidence of KRAS mutation in adenocarcinoma among the Asian population is approximately 10–15% [16]. Irreversible small-molecule KRAS G12C inhibitors have shown promising results against NSCLC [17, 18]. Currently, the standard treatments for KRAS mutated NSCLC remain chemotherapy and ICIs. In addition, NSCLC with KRAS mutations may have a better response to ICIs than KRAS negative NSCLC [19–21]. KRAS inhibitor drugs have not been widely available. For elder patients who cannot tolerate chemotherapy, immunotherapy or immunotherapy combined with anlotinib serve as alternative treatments.

The most common adverse reaction to anlotinib is hypertension. The relationship between PFS with anlotinib treatment and adverse reactions is interesting. To our knowledge, hypertension and hand-foot syndrome are the common adverse reactions to angiogenic inhibitor treatment [22, 23]. Song et al. [24] have investigated the efficacy and safety of anlotinib for elder patients with previously treated extensive-stage small cell lung cancer and the mPFS of elderly patients with and without anlotinib-induced hypertension was 4.35 and 2.95 months, respectively, and the difference was statistically significant (*P* = 0.01). They demonstrated that patients with hypertension and hand-foot syndrome might have superior prognosis. Another study found treatment-induced hypertension was a predictor only for patients without previous hypertension, who had longer PFS [25]. In our study, we also showed that the occurrence of hypertension has important significance in guiding prognostication for elder patients with EGFR negative NSCLC. However, the mechanism of the correlation between hypertension and better prognoses in patients treated with anlotinib still requires further study. Five patients stopped anlotinib treatment, and seven patients required a decreased dose because of adverse reactions. Adverse reactions were recorded according to EGFR gene status and the mode of anlotinib treatment, and no significant differences were observed. Therefore, anlotinib appears to be well tolerated in patients receiving treatment, the adverse reactions are controllable, and no significant increase in adverse reactions is seen in elder patients receiving combined treatment. In clinical treatment, close monitoring of adverse reactions and timely adjustment of dosages are necessary.

We recognize the limitations of our research, mainly the study’s retrospective nature. Because of differences in histology and genetic status, and the lack of a unified treatment model, heterogeneity and statistical differences existed in the data. This study involved anlotinib combination therapy, which must be validated in future clinical trials.

## Conclusions

Our research showed that anlotinib alone or combined with ICIs is an effective regimen for the treatment of elder patients with advanced NSCLC and is well tolerated. Moreover, anlotinib combined with EGFR-TKIs may reverse EGFR-TKI resistance. In the future, more prospective studies of multiple treatment modes should be performed to explore the efficacy and safety of different populations.

## Data Availability

The datasets generated and/or analyzed during the current study are not publicly available due to limitations of ethical approval involving the patient data and anonymity but are available from the corresponding author on reasonable request.
